# Does a look of fear prompt to act? The effects of gaze and face emotional expression on manipulable objects

**DOI:** 10.3389/fpsyg.2022.927104

**Published:** 2022-09-02

**Authors:** Elisa Scerrati, Sandro Rubichi, Cristina Iani

**Affiliations:** ^1^Department of Biomedical, Metabolic and Neural Sciences, University of Modena and Reggio Emilia, Modena, Italy; ^2^Center for Neuroscience and Neurotechnology, University of Modena and Reggio Emilia, Modena, Italy; ^3^Department of Surgery, Medicine, Dentistry and Morphological Sciences With Interest in Transplant, Oncology and Regenerative Medicine, University of Modena and Reggio Emilia, Modena, Italy

**Keywords:** handle-response compatibility, manipulable objects, gaze-cueing, face emotion, fearful faces

## Abstract

Gaze direction is an important social cue for understanding the intentions of other people. Indeed, interacting with others requires the ability to encode their current focus of attention in order to predict their future actions. Previous studies have showed that when asked to detect or identify a target, people are faster if shown a gaze oriented toward rather than away from that target. Most importantly, there is evidence that the emotion conveyed by the face with the averted gaze matters. We further tested the interplay between gaze and face emotion in the context of manipulable objects to understand whether and to what extent other people's gaze influences our own actions toward objects. Participants judged whether a target graspable object was upright or inverted after viewing a face cue with a central or averted gaze. Importantly, the target's handle could be oriented toward the gazed-at location or the opposite side such that gaze and handle were corresponding or non-corresponding in space. Furthermore, we manipulated the expression of the cue by using neutral and fearful faces. Results showed a handle-response (H-R) compatibility effect (i.e., a facilitation when the response key is on the same side as the object's handle) only with fearful cues with a central gaze.

## Introduction

Interacting with others requires to encode their current focus of attention in order to be able to predict their future actions. Furthermore, the direction of eye gaze can interact with face emotion, thus facilitating social interactions (e.g., Frischen et al., 2007). In this study, we explore attention processes activated when a gaze shift is observed and how these interact with concurrent facial expression analysis, as well as with subsequent orienting of attention to grasping actions.

### The gaze-cueing effect

In a typical gaze-cueing paradigm (Friesen and Kingstone, 1998), people are required to detect or identify a target appearing on the left or right side of the screen after being cued with a centrally displayed face with a central or averted gaze. Responses are faster when the target appears in a location consistent with gaze direction (right gaze-right target and left gaze-left target) rather than inconsistent (right gaze-left target and left gaze-right target). Studies in this tradition show that gaze direction is a powerful cue to orient attention, broadening Posner's (1980) cueing task where light flashes at the periphery were used as cues. However, in Posner's studies at short cue-target interval (i.e., 100 ms), targets presented at the previously cued location were detected faster than targets presented at previously uncued location, thus producing a facilitative effect, whereas at longer cue-target intervals (i.e., 300 ms), targets presented at the previously cued location were detected more slowly than targets presented at previously uncued location, thus producing an interference effect known as “inhibition of return” (Posner and Cohen, 1984). Conversely, in gaze-cueing paradigms, the facilitative effect is delayed even up to 700 ms (e.g., Driver et al., 1999) and there is limited evidence of inhibition of return at much longer cue-target intervals (2,400 ms; Frischen and Tipper, 2004).

Even though orienting of attention in the direction of eye gaze has been considered an automatic process, evidence accumulated in the last years shows that it may be influenced by social factors (refer to Dalmaso et al., 2020 for a review), as well as by the emotion conveyed by the face (e.g., Fox et al., 2007; Chen et al., 2021) and by prior interactions with the depicted face (e.g., Ciardo et al., 2015).

### The handle-response compatibility effect

In a typical handle-response (H-R) compatibility paradigm (e.g., Tucker and Ellis, 1998), people are required to determine the upright or inverted position of a depicted graspable object, which is displayed centrally on the screen with its handle oriented either toward the left or toward the right. Responses are faster when the handle's orientation is spatially aligned with the required response (both on the left or on the right side) rather than misaligned (handle on the left and response on the right or *via*).

According to the motor activation account (e.g., Ellis, 2018), the H-R compatibility effect reflects the activation of motor programs to interact with objects: perceiving, say, a cup's handle would activate a motor program for grasping it with the left or right hand. On the contrary, according to the location coding account (e.g., Cho and Proctor, 2010), the H-R compatibility effect indicates the activation of a location code relative to the handle: being the handle's orientation a perceptually salient feature of the object, it would activate a spatial response code.

Exploring the H-R compatibility effect in the context of the orienting of spatial attention may prove fruitful to disentangle between the two accounts.

### The present study

Our purpose is to combine the two abovementioned areas of research to test whether other people's gaze influences our own actions toward objects. Since there is evidence of enhanced gaze-cueing effects for facial expressions conveying emotions (e.g., Tipples, 2006), we will examine gaze orienting in the context of emotional facial cues. Given that literature suggests a more robust distinction between fear and neutral facial expression (e.g., Tottenham et al., 2011), we will focus on these two facial expressions.

In addition, literature suggests that it takes longer to classify peripheral target letters when fearful facial expressions are presented at fixation relative to neutral expressions (delayed disengagement hypothesis: Georgiou et al., 2005). Therefore, we will introduce cue faces with either a fearful or neutral expression looking centrally to test a potential modulation of the H-R compatibility effect due to emotion processing. Participants will judge whether a target graspable object is upright or inverted, i.e., a discrimination that is widely used to assess the H-R compatibility effect (e.g., Saccone et al., 2016; Iani et al., 2019), after being presented with a neutral or fearful cue face looking centrally or toward one or the other side. Importantly, the target's handle could be oriented toward the gazed-at location or the opposite side such that gaze and handle will be corresponding (valid gaze), non-corresponding (invalid gaze), or unmatched (central gaze) in space.

The directional gaze conveyed by the cue might generate a spatial code (left, right, and central) that is independent from the spatial code generated by the target's spatial feature (i.e., the object's handle) or that interacts with it. If the two codes are independent, responses should be faster when the direction of the gaze is consistent with the spatial location of the response key and when the direction of the target's handle is consistent with the spatial location of the response key. This result would be in line with previous findings, showing independent coding of gaze direction and stimulus spatial location (e.g., Zorzi et al., 2003; Ricciardelli et al., 2007; Villani et al., 2021), and would speak in favor of the location coding account (e.g., Cho and Proctor, 2010) since it would assimilate the H-R compatibility effect to other spatial stimulus-response (S-R) compatibility effects, such as the Simon effect (Simon, 1990).

On the contrary, if the two codes interact, support for the location coding account would be undermined as the H-R compatibility effect would not behave as other spatial compatibility effects, hence suggesting that a different mechanism underlies the effect. Specifically, if attention processes initiated by eye gaze affect interactions with objects, we expect to observe that a valid (rather than invalid) cue prepares for action with the target object, thus leading to a greater H-R compatibility effect (Ellis, 2018). In addition, if attention processes triggered by eye gaze are potentiated by emotional facial cues, we expect to observe an even greater H-R compatibility effect with fearful than neutral valid cues. However, a valid cue could also disrupt the activation of motor programs intended to interact with the object since the latter could be perceived as attended to by others and, therefore, as a “busy” object. If so, we should observe that an invalid (rather than valid) cue leads to a greater H-R compatibility effect (refer to Iani et al., 2019 for similar predictions).

Finally, if there is a specific tendency to dwell on fear-relevant stimuli (delayed disengagement hypothesis; Georgiou et al., 2005), we should observe slower responses on H-R incompatible trials with fearful central than fearful invalid cues. Indeed, a greater H-R compatibility effect with fearful than neutral cue faces looking centrally is expected as disengaging attention from fearful cue faces should take longer. As a consequence, they should impact more drastically on subsequent processing. Conversely, if a fearful straight gaze facilitates the subsequent processing of the target (facilitation hypothesis; e.g., Carlson, 2016), we should observe faster responses on H-R compatible trials with central than valid cues since there would be a facilitated orienting of spatial attention with this type of cues.

## Methods

### Participants

We calculated the sample size required to achieve 80% power to detect a significant interaction between *Gaze* (valid, invalid, and central) and *H-R compatibility* (compatible and incompatible) using G^*^power version 3.1 (Faul et al., 2007). With an effect size f = 0.1758631 (obtained from a medium ${\text{n}}_{\text{p}}^{2}$ = 0.03), the power calculation gave a recommended sample size of at least 36 participants. A total of 72 (55 women; mean age: 25 years; SD: 10 years) students from the University of Modena and Reggio Emilia took part in the experiment. They were all right-handed (laterality mean = 0.82; SD = 0.14). To note 36 (25 women; mean age: 30 years; SD: 11 years) students were randomly assigned to the neutral gaze condition, and 36 (30 women; mean age: 21 years; SD: 8 years) students were randomly assigned to the fearful gaze condition. They all had normal or corrected-to-normal vision and were naive as to the purpose of the experiment. They all served as unpaid volunteers. The experiment was conducted in accordance with the ethical standards laid down in the Declaration of Helsinki and fulfilled the ethical standard procedure recommended by the Italian Association of Psychology (AIP). All participants gave their written informed consent to participate to the study.

### Apparatus and stimuli

The study was conducted online (also refer to Dalmaso et al., 2020; Villani et al., 2021 for gaze-cueing studies conducted remotely). We used the Gorilla Experiment Builder (www.gorilla.sc) to create and host our experiment (Anwyl-Irvine et al., 2020; for a critical overview, refer to Scerrati et al., 2021). Automated procedures ensured that participants were all using a desktop computer and automatically rejected participants who took more than 2 h to complete the task. To minimize potential distractions, participants were invited to carry out the experiment in a quiet place and to avoid the manipulation of any object throughout the task. In addition, before starting, participants were asked to close background apps, softwares, and all browser windows except for that of the experiment. Photographs selected from the Karolinska Directed Emotional Faces set (KDEF; Lundqvist et al., 1998) were used for the cues. Four cue face stimuli (2 men and 2 women) were used in the neutral condition and 2 cue face stimuli (1 men and 1 women) were used in the fearful condition.^1^ Three versions of each face stimulus were used; the central gaze version was available from the KDEF; two additional versions, one gazing left and one gazing right, were taken from Ricciardelli et al. (2012)^2^. See the Appendix for an overview of cue stimuli.

The target stimulus was the photograph of a cup made of plastic taken by Scerrati et al. (2020a), Experiment 2 who demonstrated a critical role of the functional part of the object in the occurrence of the H-R compatibility effect. An inverted version of the cup was digitally generated by a mirror reversal on the vertical axis by using Gimp 2. Both the cue and the target photographs were rendered in greyscale.

Responses were emitted by pressing the “e” (left) and “o” (right) keys on a QWERTY keyboard if the keyboard lacked the numeric pad, and the “y” (left) and “p” (right) keys if the keyboard had the numeric pad.^3^

### Procedure

Participants were requested to discriminate the upright/inverted position of the target stimulus on the monitor as rapidly and accurately as possible. Half of the participants in each experimental condition (neutral cues and fearful cues) pressed the E/Y-key on the computer keyboard with their left index finger to indicate an upright target, and the O/P-key with their right index finger to indicate an inverted target. The other half was assigned to the opposite mapping. Emotion expressions of the faces (neutral and fearful) were manipulated between participants to avoid the potential carry-over effect of the fearful faces over the neutral ones and/or viceversa. Indeed, there is evidence that participants are more accurate at categorizing/evaluating faces when presented with only one emotional expression at a time. For instance, Ricciardelli et al. (2016) found that neutral facial expressions were perceived and categorized as negative and grouped together with angry and fearful faces when the three different emotions were presented intermixed in the same block.

**Figure 1:**
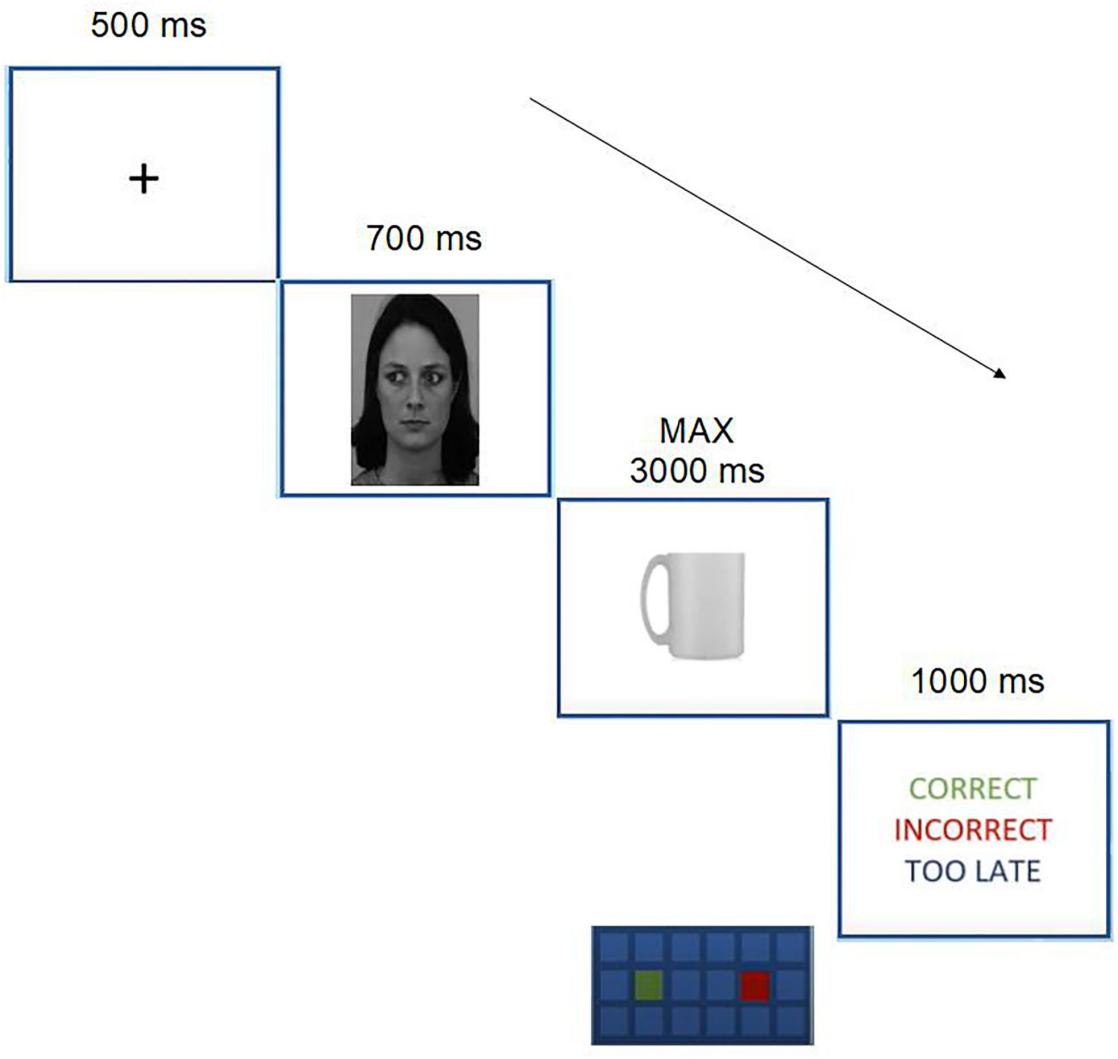
Sequence of events in the neutral condition. In the example above, the cue was valid, and instructions required to respond with the left index finger to upright objects and with the right index finger to inverted objects. Note that elements are not drawn to scale. Facial stimuli reproduced with permission from the KDEF stimulus set, available at (https://kdef.se/download-2/register.html). The image ID depicted in the Figure is AF01NES.

The sequence of events on each trial was as follows. A black fixation cross (0.5 × 0.5 cm) appeared on a white background at the center of the screen for 500 ms. This was then replaced by a face cue, which was equally likely to gaze centrally, toward the left or toward the right, and stayed on screen for 700 ms.^4^ Then, the target object appeared centrally with the handle facing left or right and remained on the screen until the participant's response or 3,000 ms had elapsed. Participants' response triggered feedback (“Correct,” “Incorrect,” or “Too slow”; on the importance of feedback for online experiments, refer to Sauter et al., 2020) which appeared in red on a white background for 1,000 ms, depending on the response accuracy and speed (see Figure 1 for a schematic representation of the sequence of events), and then the whole cycle of events was repeated to produce the next trial.

Participants performed 24 practice trials followed by four blocks of 48 trials each for a total of 216 trials per participant. The order of trials within each block was randomly determined. Blocks were separated by a self-paced interval and the experiment lasted for approximately 20 min.

## Results

Omissions (0.06%), incorrect responses (9.31%), and response times (RTs) faster/slower than the overall participant's mean minus/plus 3 SD (3.36%) were excluded from the analyses.

Two repeated-measures ANOVAs with *Gaze* (valid, invalid, and central) and *H-R compatibility* (compatible and incompatible) as within-subject factors, and *Condition* (neutral cues and fearful cues) as the between-subject factor^5^ were conducted separately on RTs and arcsine-transformed error rates.

### RTs

The main effects of *Gaze* and *Condition* were non-significant, *F*(2,140) = 1.15, *p* = 0.31, $n_{p}^{2}$ = 0.01; *F*(1,70) = 2.89, *p* = 0.09, $n_{p}^{2}$ = 0.04, respectively, whereas *H-R compatibility* was significant, *F*(1,70) = 4.57, *p* = 0.03, $n_{p}^{2}$ = 0.06, with slightly faster response latencies for H-R compatible (M: 577 ms; standard error [SE]: 8.9) than incompatible (M: 583 ms; SE: 9.8) trials. Crucially, the three-way interaction between *Gaze, H-R compatibility*, and *Condition* was significant, *F*(2,140) = 5.15, *p* = 0.007, $n_{p}^{2}$ = 0.06. No other significant interactions were observed, *F*_*s*_ <1.

Given the three-way significant interaction between *Gaze, H-R compatibility*, and *Condition*, we conducted separate ANOVAs for each level of *Condition* (neutral cues and fearful cues). Neutral cues did not highlight a significant main effect of *H-R compatibility* or *Gaze* nor their interaction, *F*_*s*_< 2.03, *p*_*s*_ > 0.13; $n_{p}^{2}$ < 0.05. On the contrary, fearful cues highlighted a main effect of *H-R compatibility, F*(1,35) = 4.15, *p* = 0.04, $n_{p}^{2}$ = 0.10, with slightly faster response latencies for H-R compatible (M: 560 ms; SE: 11.3) than incompatible (M: 568 ms; SE: 13.1) trials. The main effect of Gaze was non-significant, *F*(2,70) = 1.71, *p* = 0.187, $n_{p}^{2}$ = 0.04. Importantly, there was a significant interaction between *H-R compatibility* and *Gaze, F*(2,70) = 3.21, *p* = 0.04, $n_{p}^{2}$ = 0.08. The Bonferroni-corrected planned comparisons showed that responses were slower with H-R incompatible trials when these were preceded by a central (M: 578 ms, SE: 13.0) rather than invalid (M: 562 ms, SE: 13.2) gaze cue, *t*(35) = 3.00, *p* = 0.005.

**Figure 2:**
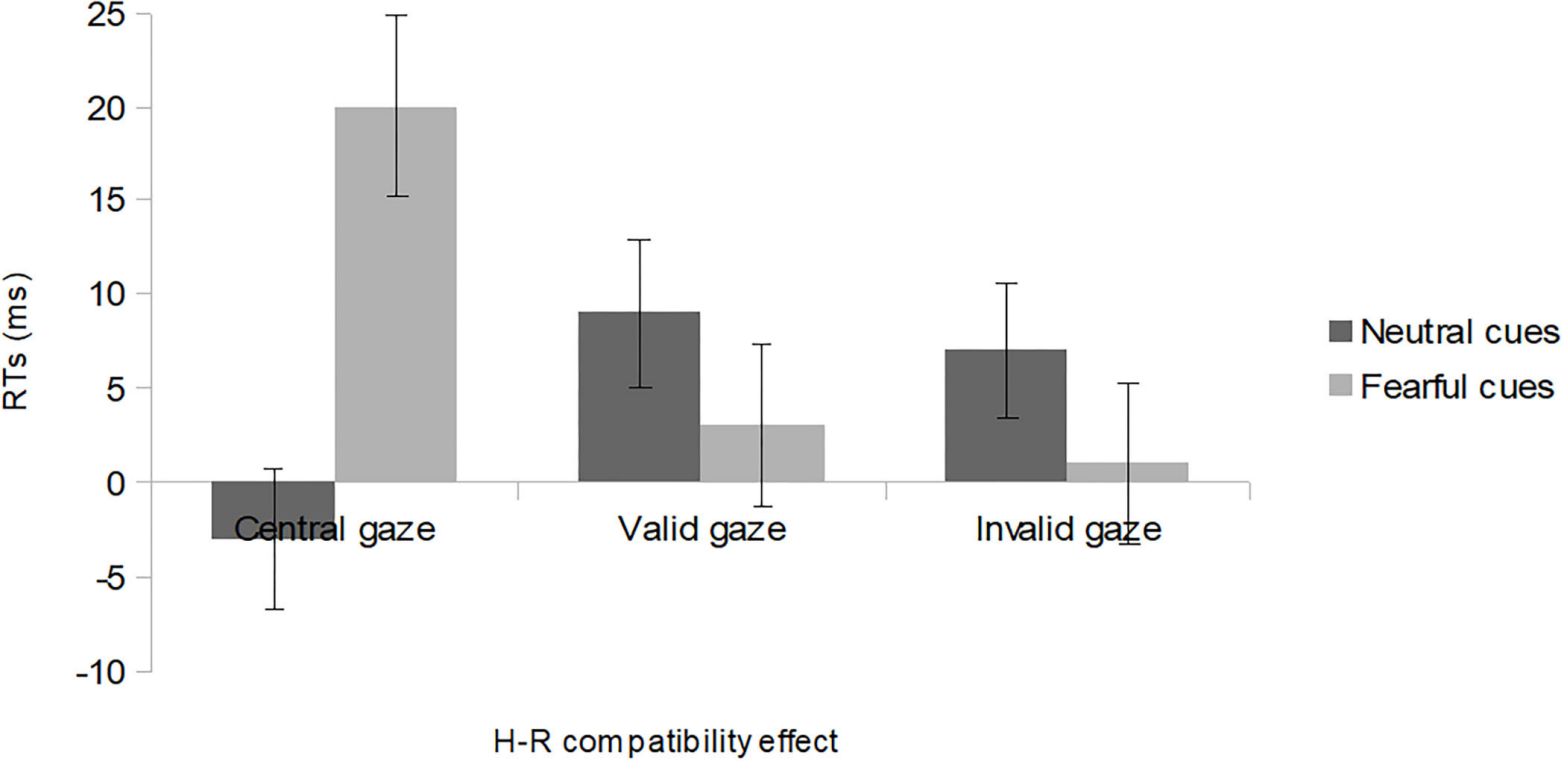
The H-R compatibility effect (mean reaction time on incompatible trials minus mean reaction time on compatible trials) for central, valid, and invalid gaze trials in both neutral and fearful cues conditions: bars indicate standard errors corrected for within-participants designs (Loftus and Masson, 1994).

In addition, we conducted the Bonferroni-corrected pairwise comparisons aimed at testing the magnitude of the H-R compatibility effect (incompatible–compatible trials) at each level of *Gaze* (valid, invalid, and central) and found that the H-R compatibility effect was significant with fearful cue faces with a central gaze, *t*(35) = 3.15, *p* = 0.003 (refer to Figure 2 for details).

### Error rates

**Figure 3:**
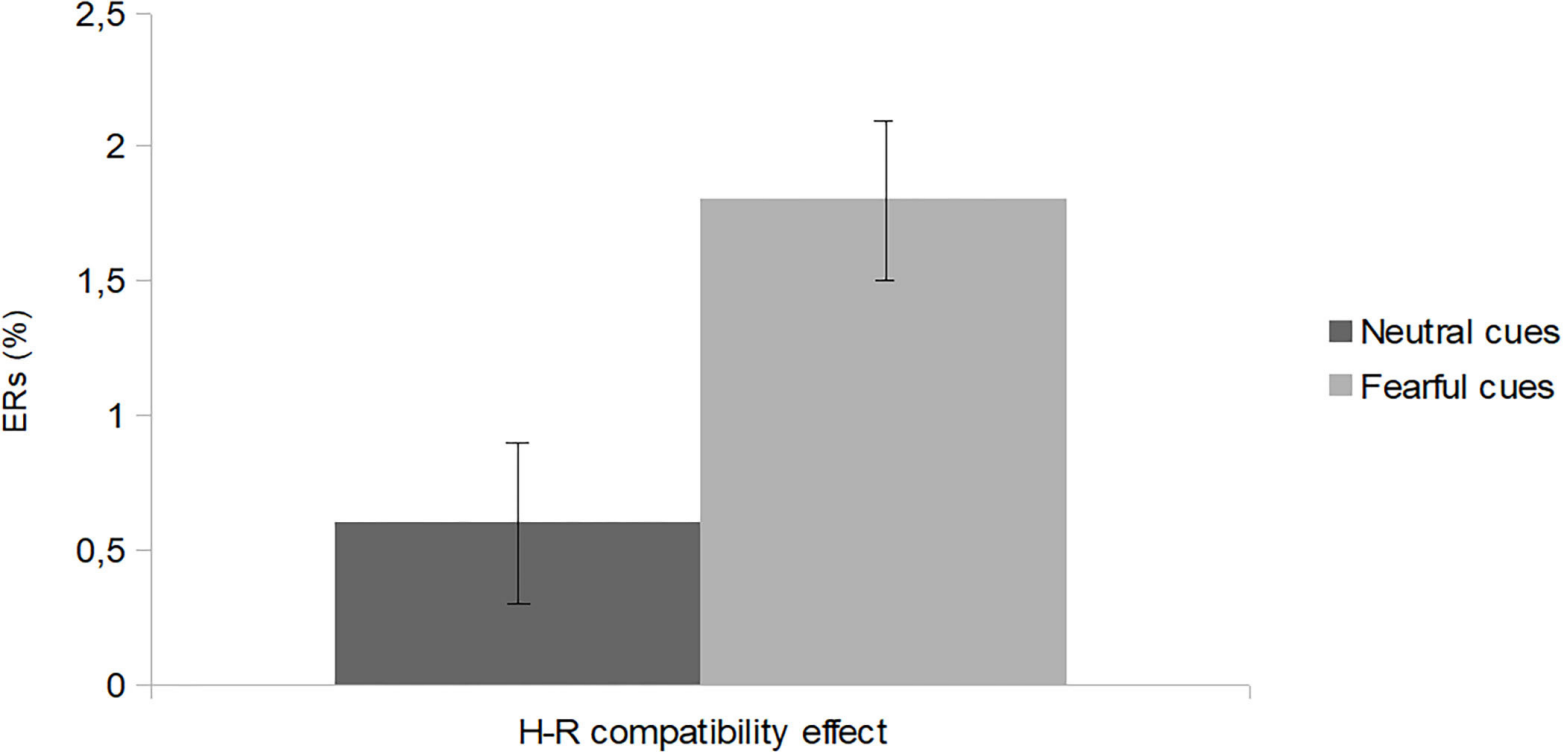
The H-R compatibility effect (mean percentage errors on incompatible trials minus mean percentage errors on compatible trials) for neutral and fearful cues conditions: bars indicate standard errors corrected for within-participants designs (Loftus and Masson, 1994).

The ANOVA on arcsine-transformed error rates revealed a main effect of *Condition*,^6^ *F*(1,70) = 4.49, *p* = 0.03, $n_{p}^{2}$ = 0.06, with lower percentage of error for neutral (M: 3.7%; SE: 0.6) than fearful cues (M: 5.5%; SE: 0.6), and a main effect of *H-R compatibility, F*(1,70) = 5.68, *p* = 0.02, $n_{p}^{2}$ = 0.07, with lower percentage of error for H-R compatible (M: 4.0%; SE: 0.4) than incompatible (M: 5.2%; SE: 0.5) trials. The main effect of *Gaze* was non-significant, *F*(2,140) = 0.87, *p* = 0.41, $n_{p}^{2}$ = 0.01. Finally, there was a marginally significant interaction between *H-R compatibility* and *Condition, F*(1,70) = 3.97, *p* = 0.05, $n_{p}^{2}$ = 0.05, indicating people were more prone to make mistakes with H-R incompatible trials in the fearful (M: 6.4%; SE: 0.8%) than neutral (M: 4.0%; SE: 0.7%) condition, *t*(70) = 2.18, *p* = 0.032. No other interaction turned out to be significant, *F*_*s*_ <1.50, *p*_*s*_ > 0.22, $n_{p}^{2}$ < 0.02. Pairwise comparisons aimed at testing the H-R compatibility effect (incompatible–compatible trials) in each *Condition* (neutral cues and fearful cues) revealed that the H-R compatibility effect was significant with fearful cue faces, *t*(35) = 2.40, *p* = 0.02, but not with neutral cue faces, *p* =0.31 (refer to Figure 3 for details).

## Discussion

This study tested whether other people's eye gaze and face emotion influence our own actions toward objects. Importantly, we found a significant H-R compatibility effect of 20 ms only with fearful cue faces looking centrally, whereas the H-R compatibility effect was disrupted by all other types of cue faces. This finding is in line with the claim that there is a specific tendency to dwell on fear-relevant stimuli (delayed disengagement hypothesis; Georgiou et al., 2005) while contrasting with a facilitation hypothesis of forward gaze (Carlson, 2016). Indeed, the H-R compatibility effect with fearful cue faces looking centrally was due to a slowing down of responses for H-R incompatible trials rather than to a speed-up of responses for H-R compatible trials. Thus, being alerted about a potential threat in the environment by a central fearful gaze may capture our attention to a higher degree than a central neutral gaze since it may prompt people to act (refer also to Scerrati et al., 2022 for action under threatening circumstances).

It is worth noting that, unlike previous studies showing independent coding of gaze direction and stimulus spatial location (e.g., Zorzi et al., 2003; Ricciardelli et al., 2007; Villani et al., 2021), we did not find additive effects of gaze direction and H-R compatibility, neither for RTs nor for error rates. Therefore, it seems that the stimulus spatial feature (i.e., the orientation of the object's handle) does not behave as the stimulus spatial location, hence, undermining the location coding account's assumption that the H-R compatibility effect is a kind of S-R compatibility effect (e.g., a Simon effect: Cho and Proctor, 2010).

Contrary to our expectations, no significant difference in the H-R compatibility effect was observed between the valid and invalid gaze conditions, not even when using fearful facial cues. This result might stem from at least two reasons, which are not mutually exclusive. On the one hand, while in studies investigating the gaze-cueing effect, the face cue gazing left or right remained present along with the target letter until the participant's response, in our study, it was replaced by the target object after 700 ms of exposure. On the other hand, although in gaze-cueing paradigms, the facilitative effect is most pronounced at the 700 ms of cue-target interval (e.g., Driver et al., 1999; Experiment 2; refer to Dalmaso et al., 2020 for review), it is likely that with our modified paradigm, where the cue is absent during processing of the target, the facilitative effect declines before. This second explanation seems to be supported by a parallel experiment conducted in our lab where we found an H-R compatibility effect of 18 ms (p < 0.001) with neutral valid cues at 100 ms of cue-target interval (unpublished result). Therefore, the absence of the cue during the processing of the target together with the relatively long cue-target interval might explain why we failed to observe a greater H-R compatibility effect with valid than invalid cues in this study. Of course, these tentative explanations need to be further investigated. An alternative explanation may be that the cue-target interval used in this study (i.e., 700 ms) was too short to allow people to integrate gaze and emotion information from the cue face. Indeed, the process of encoding both gaze direction and face emotion from the cue face may require time as suggested by previous studies (e.g., Graham et al., 2010). In particular, this would explain why we did not observe a greater H-R compatibility effect with valid cues, not even in the fearful condition, which should be the more likely condition to expect gazing effects from, since fearful cues with an averted gaze should act more effectively as a signal of the location of potential danger (Graham et al., 2010), hence, encouraging people to orient their attention accordingly (Adams and Kleck, 2005). Importantly, our results are in line with a recent finding by Pittig et al. (2022) who found that fearful faces with averted gaze were not strong enough to create an averted gaze advantage.

It is also worth noting that results did not show a greater H-R compatibility effect with invalid than valid cues, which suggest people did not perceive the valid gaze as attending to the target object as per interacting with it and, therefore, the target object as a “busy” object. This result is in line with previous studies (Iani et al., 2019; Scerrati et al., 2019, 2020b), showing that grasped objects do not seem to be perceived as “busy” objects, i.e., objects the observer is prevented to act upon. Therefore, grasped and attended to objects may be processed similarly.

It is also worth discussing that results concerning error rates slightly differed from results concerning RTs. Indeed, while both dependent variables showed a main effect of H-R compatibility and no effect of Gaze, errors were fewer with neutral than fearful cue faces, a result that might indicate a greater propensity to make a mistake when action is prompted by a look of fear since, when exposed to threats, individuals may adopt a defensive emotional state (e.g., Pereira et al., 2006), which may increase anxiety that leads to errors. In addition, error rates also showed that the H-R compatibility effect was greater with fearful than neutral cue faces. This result was due to people making mistakes more in the fearful than neutral condition with H-R incompatible trials, a finding that suggests that although a look of fear may prompt to act, people are liable to activate the incorrect action when there are threats around them and the responding hand and target spatial feature are misaligned.

One limitation of our study is that we recruited an unselected sample of people for whom we do not know the level of anxiety. Future studies may look further into the impact of attention processes triggered by gaze shift potentiated by emotional facial expressions on the processing of actions by using a selected sample of participants with trait anxiety.

A further limitation of this study is that whether the face conveys a neutral or a fearful expression has been manipulated between-subject. Indeed, this may have decreased the power of the study; hence, a replication with a within-subject design seems desirable.

Finally, it is worth emphasizing that our findings were obtained with a discrimination task (i.e., orientation judgement) typical in the study of the H-R compatibility effect but atypical in the context of gaze-cueing effects. Therefore, future studies may deepen the investigation of the interactions between the two phenomena by adopting tasks more suitable for the occurrence of gaze-cueing effects.

In conclusion, our study seems to suggest that eye gaze and facial expression may act as moderators of the H-R compatibility effect with the effect appearing when fearful facial expression with a central gaze is presented as cues and disappearing when neutral facial expressions with all three gaze directions (valid, invalid, and central) are presented as cues, and when fearful facial expressions with both valid and invalid eye gaze are presented as cues. This result suggests a specific role of fearful facial expression with central gaze in the occurrence of the H-R compatibility effect likely signaling a strong engagement of covert/overt attention may be needed for the activation of motor programs and the encoding of spatial information.

## Data availability statement

The original contributions presented in the study are included in the article/Supplementary material, further inquiries can be directed to the corresponding author/s.

## Ethics statement

Ethical review and approval was not required for the study on human participants in accordance with the local legislation and institutional requirements. The patients/participants provided their written informed consent to participate in this study.

## Author contributions

ES: conceptualization, methodology, software, data collection, data analyses, and writing—original draft preparation. SR: conceptualization, methodology, and supervision. CI: conceptualization, methodology, data analyses, writing—reviewing and editing, supervision, and funding. All authors contributed to the article and approved the submitted version.

## Conflict of interest

The authors declare that the research was conducted in the absence of any commercial or financial relationships that could be construed as a potential conflict of interest.

## Publisher's note

All claims expressed in this article are solely those of the authors and do not necessarily represent those of their affiliated organizations, or those of the publisher, the editors and the reviewers. Any product that may be evaluated in this article, or claim that may be made by its manufacturer, is not guaranteed or endorsed by the publisher.

## Figures and Tables

**Figure A1:**
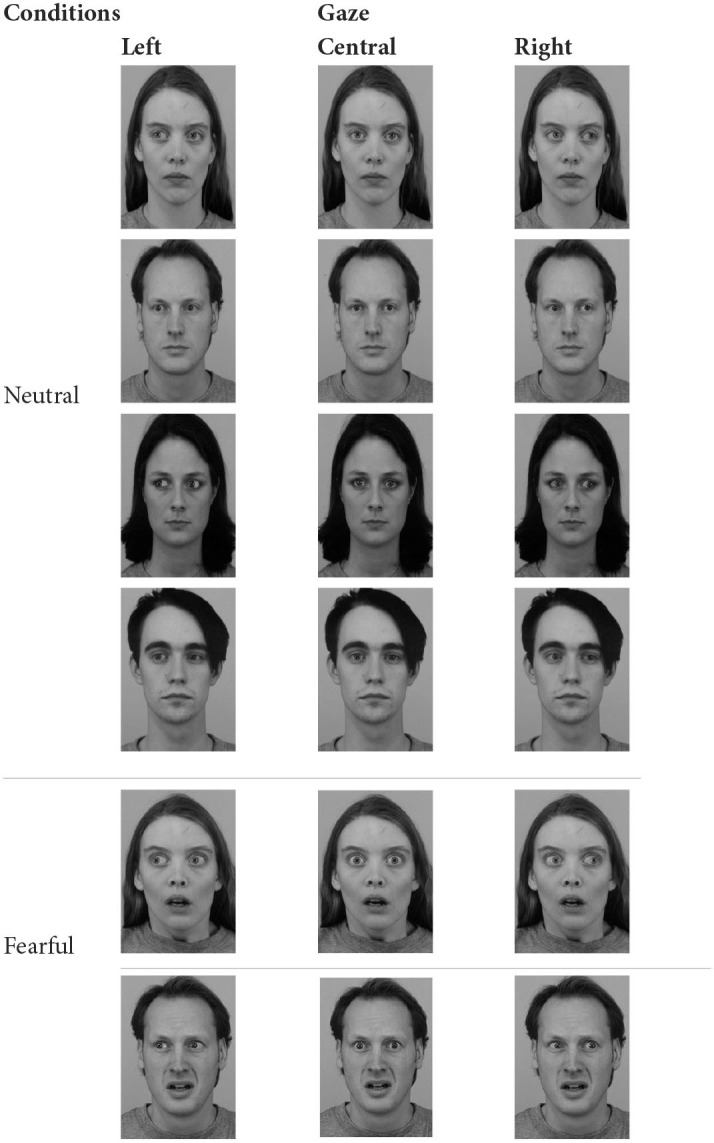
Cue stimuli used in the neutral and fearful conditions. Facial stimuli reproduced with permission from the KDEF stimulus set, available at (https://kdef.se/download-2/register.html). The KDEF IDs of the stimuli used in the experiment (and depicted below) are AF01NES, AF21NES, AF21AFS, AM10NES, AM10AFS, AM24NES.

## Footnotes

^1^After completion of the Experiment, we discovered that we had inadvertently included only 2 (1 male and 1 female) out of 4 (2 males and 2 females) face identities in the fearful condition.

^2^Upon completion of the main Experiment, people were asked to rate the seen cue faces with both the averted (left, right) and the central gaze as for the emotion conveyed (i.e., anger, disgust, happiness, neutral, sadness, and fear) on a 5-point Likert scale (1 = not at all, 5 = extremely). Pairwise comparisons revealed that in the neutral condition, the mean rating scores were higher for the neutral emotion (3.2) compared to the others (1.4 on average), *t*_*s*_(35) >, 6.14 *p*_*s*_ < 0.001, indicating that the emotion conveyed by the face was clearly recognisable as a neutral one. Similarly, in the fearful condition, the mean rating scores were higher for the fearful emotion (4.0) compared to the others (1.4 on average), *t*_*s*_(35) >, 15.20 *p*_*s*_ < 0.001, indicating that the emotion conveyed by the face was clearly recognisable as a fearful one.

^3^This double response arrangement was aimed at ensuring participants could code response keys location with their left/right hemifield since keyboards could vary across participants given the experiment was web-based. We would like to highlight that in either condition (fearful, neutral) participants with the numeric pad on the right were 20 out of 36, therefore there is little chance this aspect affected our results (i.e., the occurrence of an interaction between gaze-cueing and H-R compatibility only in the fearful condition). To further examine this possibility, we conducted a mixed ANOVA with Gaze (valid, invalid, central) and H-R compatibility (compatible, incompatible) as within-subject factors, and Condition (neutral cues, fearful cues) and Numeric Pad (present, absent) as between-subject factors. The main effect of Numeric Pad was not significant, *F* = 0.254, *p* = 0.616. In addition, Numeric Pad did not interact with any of the other factors, *F*_s_ <0.220, *p*_s_ > 0.142.

^4^Importantly, an enhanced GCE for fearful faces has been observed in studies using SOAs in the 160–700 ms range (e.g., Uono et al., 2009; Graham et al., 2010; Bayless et al., 2011; McCrackin and Itier, 2018; Liu et al., 2019; see Dalmaso et al., 2020 for review).

^5^Previous research mainly manipulated the emotion conveyed by the cue face within-participants. In this study we manipulated it between-participants. Therefore, there is a chance that the two groups ensuing from the emotion manipulation could be confounded on some other variable (e.g., age, gender). To exclude this possibility we conducted an ANOVA where age and gender were included as covariates. There was niether a significant main effect of age (*p* = 0.124) nor of gender (0.101). In addition, age did not interact with Gaze (*p* = 0.406) and H-R compatibility (*p* = 0.667), nor there was a significant three-way interaction of these variables (*p* = 0.911). Similarly, gender did not interact with Gaze (*p* = 0.934) and H-R compatibility (*p* = 0.955), nor there was a significant three-way interaction of these variables (*p* = 0.540). Importantly, the three-way interaction of Gaze (valid, invalid, central), H-R compatibility (compatible, incompatible) and Condition (neutral, fearful) was significant even when age and gender were included in the analysis, *F*(2,136) = 3.73, *p* = 0.02, $n_{p}^{2}$ = 0.05. These results indicate that age and gender did not influence our results, strengthening our findings obtained with the between manipulation.

^6^We feel confident that having 2 rather than 4 face identities in the fearful and neutral condition, respectively, did not influence the results since we did not obtain a main effect of Condition for RTs (*p* = 0.09). Although we did observe a main effect of Condition for ERs, there was a lower percentage of error for neutral (M: 3.7%; SE: 0.6) than fearful cues (M: 5.5%; SE: 0.6), indicating that participants' performance was not worsened when processing a larger number of face identities (i.e., in the neutral condition). In addition, a further ANOVA with *Cue identity* as a within-subject factor did not show a main effect of this variable (*p* =.495), nor any significant interaction involving it (*F*_*s*_ <0.160 *p*_*s*_ > 0.148).
